# Supplementary material for: Dynamically-enhanced strain in atomically thin resonators
Source: Nat Commun. 2020 Nov 2;11:5526. doi: 10.1038/s41467-020-19261-3 (PMC7608634; doi:10.1038/s41467-020-19261-3)
Supplement: Supplementary file 1 — Supplementary Information [file 41467_2020_19261_MOESM1_ESM.pdf]

Supplementary Information for:

Dynamically-enhanced strain in atomically thin resonators

Xin Zhang,* Kevin Makles, Léo Colombier, Dominik Metten,

Hicham Majjad, Pierre Verlot, and Stéphane Berciaud†

* zhxsemi@gmail.com

† stephane.berciaud@ipcms.unistra.fr

CONTENTS

Supplementary Note 1. Raman scattering in graphene 3

Supplementary Note 2. Sample design and interference effects 7

Supplementary Note 3. Elementary modelling of static strain 9

Supplementary Note 4. Static displacement and equilibrium position shift 11

Supplementary Note 5. Displacement calibration 15

Supplementary Note 6. Mechanical response of driven graphene drums 20

Supplementary Note 7. Dynamical strain and non-linearities 24

Supplementary Note 8. Effect of laser-induced heating 27

Supplementary Note 9. Supplementary data on device 1 29

Supplementary Note 10. Supplementary data on device 2 32

Supplementary Note 11. Supplementary data on device 3 36

Supplementary References 38

This Supplementary Information file is organised as follows. In Supplementary Note 1, we provide details on the Raman scattering response of graphene and on our fitting procedure. In Supplementary Note 2 and Supplementary Note 3, we outline the sample design and discuss an elementary mechanical model, respectively, before discussing, in Supplementary Note 4, how optical interference effects allow estimating the static displacement ξ of a graphene drum and the static strain ε_{s} it undergoes. In Supplementary Note 5, we present a comprehensive displacement calibration scheme using three different methods that yield a consistent and accurate determination of the root mean square (RMS) displacement z_{rms} in the driven regime. These results also allow us to conclude that, within experimental accuracy, the effective mass of our drum is that of a pristine graphene monolayer. In Supplementary Note 6, we present a basic modelling of the mechanical response of graphene both in the linear and non-linear regime, followed by a discussion on the links between dynamical strain and non-linearities in Supplementary Note 7. Laser-induced heating effects are addressed in Supplementary Note 8. Finally, supplementary data on devices 1, 2 and 3 are presented in Supplementary Notes 9, 10 and 11, respectively. This material complements and/or bolster the data shown in the main text. Devices 1, 2 and 3 have similar designs.

Supplementary Note 1. Raman scattering in graphene

The G mode and the 2D mode

As introduced in the main text, our study focuses on the well-documented G mode and 2D modes in graphene[1]. Simplified sketches of the G- and 2D-mode processes are shown in Supplementary Fig. **1**. The G mode is a one phonon non-resonant process originating from in-plane (LO and TO) zero momentum optical phonons, that is at the centre (Γ point) of the Brillouin zone. The G-mode feature is commonly described as a single, quasi-Lorentzian feature[2]. The 2D-mode is a resonant, symmetry allowed two-phonon process involving a pair of near-zone edge TO phonons near the edges of the Brillouin zone (K and K′ points)[3–5]. This 2D-mode frequency depends both on the electronic and phononic dispersion and hence on the incoming laser photon energy. The 2D mode-lineshape is *a priori* very complex[4]. In the case of suspended graphene, this lineshape is phenomenologically fit to the sum of two modified Lorentzian profiles, as in Supplementary Ref. 6.

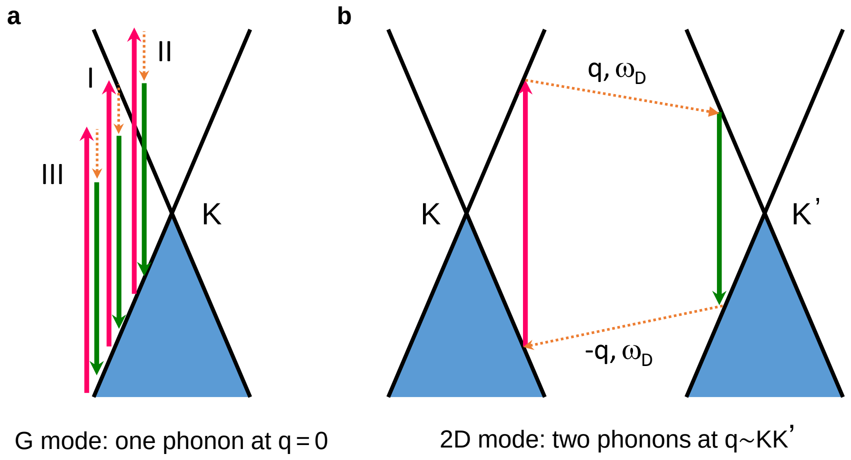

Supplementary Figure 1. **Raman scattering processes in graphene.** The pink, green and dashed orange arrows in **a** and **b** indicate incoming photons, scattered photons and scattered phonons, respectively. The G mode (**a**) is a one-phonon process involving zone-center optical phonons (LO and TO)[1]. Although resonant processes (I) may contribute to the G-mode intensity, the G-mode feature arises for the most part from the quantum interference between non-resonant processes (II, III) across the whole Brillouin zone[7]. The 2D mode (**b**) is a resonant inter-valley process involving a pair of near zone-edge TO phonons with opposite momenta $\pm q$. Here, for clarity, we only represent the so-called inner process involving phonons with momenta smaller than KK′ (Supplementary Ref. 5 and 6).

Fitting the Raman 2D-mode spectra

As discussed above and in Supplementary Ref. 6, the 2D-mode lineshape in suspended graphene is asymmetric and best fit with the sum of two modified Lorentzian profiles, as exemplified in Supplementary Fig. **2** and in Fig. 2b and 3b. The 2D-mode frequency ω_{2D} discussed in the main manuscript refers to the more intense $2D^-$ sub-feature unless otherwise specified (Supplementary Fig. **3**), while the 2D-mode intensity I_{2D} refers to the *total* integrated intensity of both $2D^-$ and $2D^+$ sub-features. As we show in Supplementary Fig. **3**, both low- and high-frequency 2D-mode sub-features are similarly affected in the driven regime.

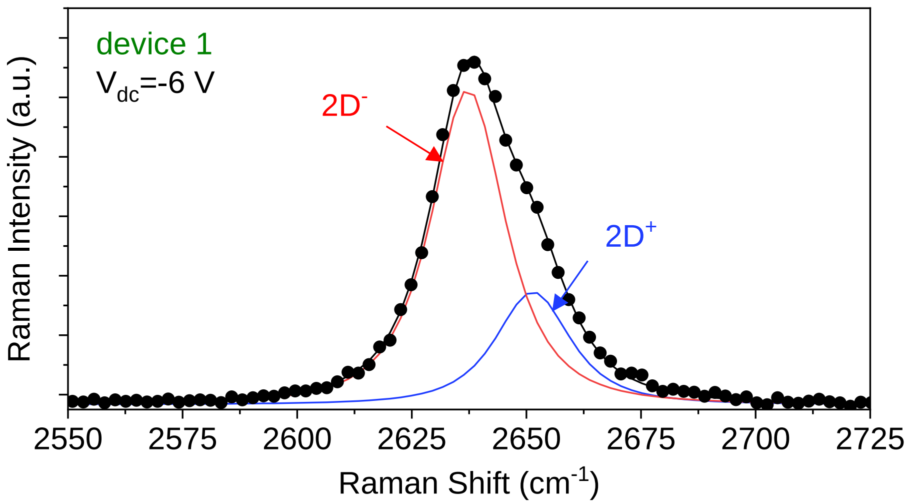

Supplementary Figure 2. **Fitting the Raman 2D-mode spectra in a suspended graphene drum**. Modified Lorentzian fit of the 2D-mode feature in suspended graphene using two sub-features, denoted $2D^-$ and $2D^+$, as in Supplementary Ref. 6.

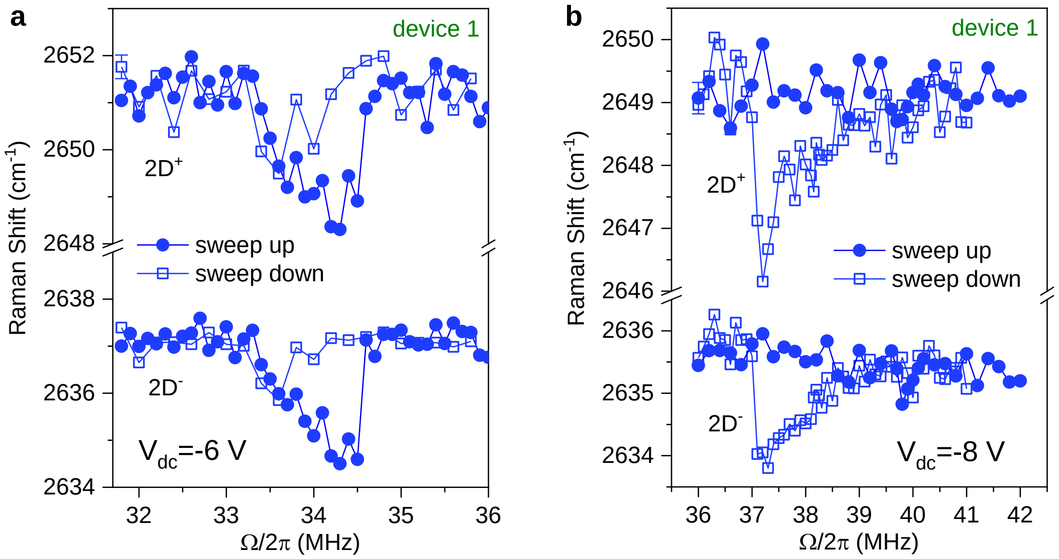

Supplementary Figure 3. **Fitting the Raman 2D-mode spectra in a resonantly-driven graphene drum**. Frequency of the two 2D mode subfeatures (ω_{2D^+} and ω_{2D^-}) as a function of the drive frequency $\Omega/2\pi$ for both upward (circles) and downward (squares) drive frequency sweeps at $V_{dc} = -8$ V (**a**) and $V_{dc} = -6$ V (**b**) in device 1 (see also Supplementary Fig. **14** and Fig. 3, respectively). Only one error bar is included in each plot for clarity.

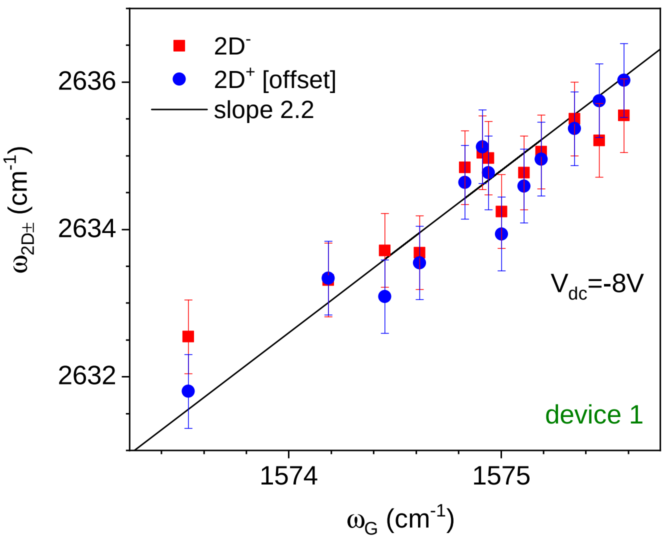

Supplementary Figure 4. **Correlation between the G- and 2D-mode frequencies**. This correlation plot is similar to Fig. 2c, except that the two components ($2D^{\pm}$) are shown. The $2D^{+}$ component is offset by $-13.7\ \mathrm{cm}^{-1}$ for a clearer comparison. The straight black line with a slope of 2.2 is a guide to the eye showing the expected correlation for strain-induced phonon softening. The $2D^{-}$ feature deviates slightly from this slope at large drive while the $2D^{+}$ feature remains closer to guide to the eye. In spite of these slight deviations, the slopes $\frac{\partial \omega_{2D\pm}}{\partial \omega_G}$ remain close to the value expected under biaxial strain.

Supplementary Note 2. Sample design and interference effects

Figure **5**a shows the vacuum/graphene/vacuum/SiO_2/Si multilayered system discussed in the main text. Due to optical interference effects, the reflectance and Raman scattering intensity depend on the laser wavelength, hole depth (d_{vac}) and residual SiO_2 thickness ($d_{\mathrm{SiO_2}}$). Starting from a given sample geometry, we have used well-established models to compute the interference enhancement factors allowing to quantitatively predict the dependence of the sample reflectance[8,9] and Raman scattered intensity[10–12] as a function of the deflection of the graphene layer, denoted ξ. As we shall see in Supplementary Note 4 and Supplementary Note 5, this modelling will allow us to accurately determine ξ in graphene drums and to calibrate displacements in the driven regime.

We have optimized the sample geometry to provide both large transduction coefficient for displacement readout (see Methods) and sufficiently intense Raman scattering signal. First, 285nm-SiO_2/Si (p-doped) substrates are chosen to easily locate monolayer graphene flakes by optical microscopy[8]. With $d_{\mathrm{vac}} = 250 \pm 5$ nm (correspondingly, $d_{\mathrm{SiO_2}}$=35 nm), the optical reflectance varies quasi-linearly with the static deflection of the membrane ξ over the range ξ=30-100 nm, ensuring a constant transduction coefficient for optical readout of the root mean square (RMS) mechanical displacement around an equilibrium position (Supplementary Fig. **5**b). At the same time, optical interferences lead to large enough Raman intensities, as shown in the calculated Raman enhancement factors[10–12] in Supplementary Fig. **5**c,d. Third, the hole diameters $2a = 5$ μm and 6 μm, are chosen such that the resonance frequency of the fundamental flexural mode (Supplementary Note 6) lies within the 50 MHz bandwidth of our detection setup.

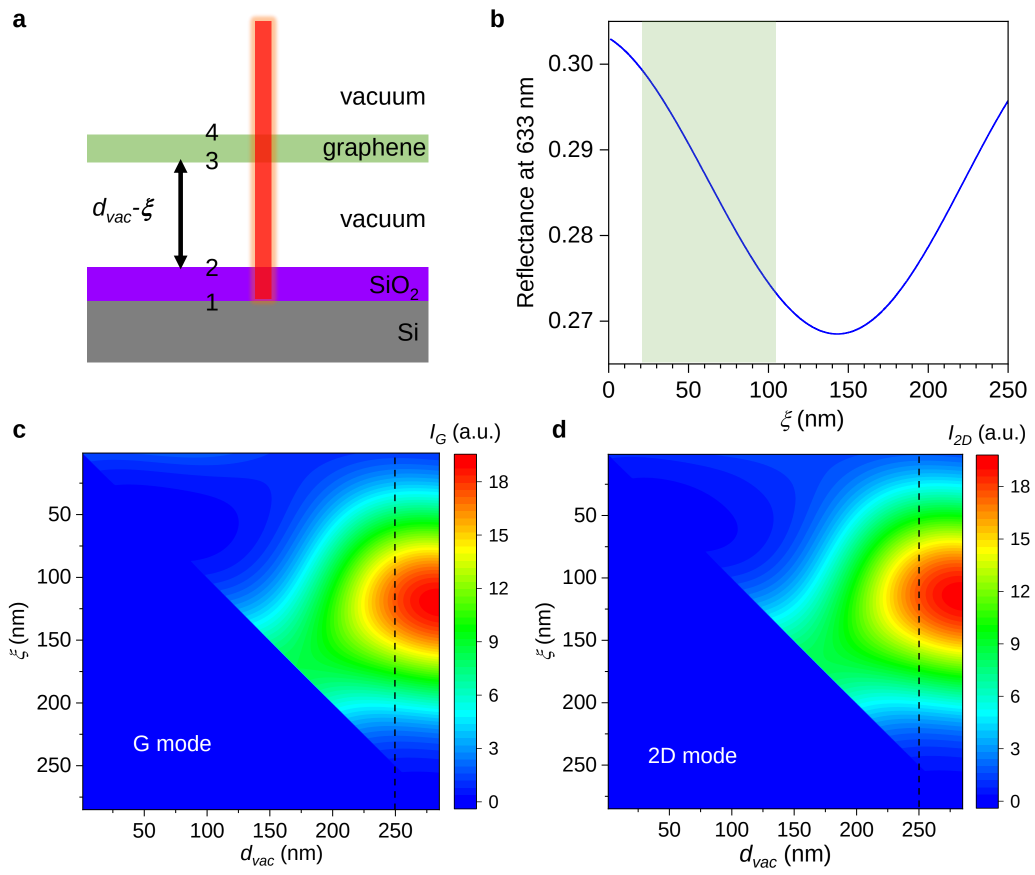

Supplementary Figure 5. Sample geometry and interference effects. a, Multilayer model for our devices, where the labels $1-4$ represent the interfaces in the system. d_{vac}-ξ is the gap between suspended graphene, displaced by ξ using a DC gate bias, and the SiO_2 surface. **b,** Calculated reflectance as a function of ξ in the case of a monolayer graphene for $d_{\mathrm{vac}} = 250$ nm and a laser wavelength of 632.8 nm. The light-green area denotes the linear region, where a large and constant transduction coefficient allows interferometric readout of the mechanical vibrations. At a small ξ, the reflectance is close to a maximum, resulting in a sharp decrease of the transduction coefficient. **c,d,** Contour plots of the G- and 2D-mode intensity enhancement factors (Supplementary Note 4) as a function of d_{vac} and ξ under optical excitation at 632.8 nm. The SiO_2 thickness is 285 nm. The black dashed lines highlight the results at $d_{\mathrm{vac}} = 250$ nm.

Supplementary Note 3. Elementary modelling of static strain

Given the radial symmetry of our system, we will consider, for the sake of simplicity, a one-dimensional model system, of a doubly clamped beam (in the membrane limit) with cross-sectional area A and length $L = 2a$. We denote x the longitudinal coordinate, with $x = 0$ corresponding to the middle of the beam. This model can be generalized to the case of a circular membrane of radius a as in Supplementary Ref. 13. We assume that under an electrostatic pressure (here, a finite gate bias V_{dc}), the membrane adopts a parabolic profile[12,14]. The downward deflection $\xi(x)$ thus writes:

$$\xi(x) = \xi(0)\left(1 - \frac{x^2}{a^2}\right), \tag{S1}$$

where $\xi(0)$ is the static deflection at the membrane's center ($x = 0$).

The elongation ΔL is:

$$\Delta L = \int_{-a}^{a} \sqrt{1 + [\xi'(x)]^2} dx - 2a \tag{S2}$$

For small deflections, *i.e.*, $\xi(x) \ll a$, the static strain ε_{s} writes:

$$\varepsilon_{\mathrm{s}} = \frac{\Delta L}{2a} = \frac{2}{3}\left(\frac{\xi}{a}\right)^2. \tag{S3}$$

In Eq. (S3) and in the following, $\xi(0)$ will be denoted ξ for simplicity.

Besides, under biaxial strain, the Raman frequency shift ($\Delta\omega_i$, with $i =$ G, 2D) relative to the unperturbed values $\omega_{i,0}$ are linked to ε_{s} by[11,15]:

$$\Delta\omega_i = 2\gamma_i\, \varepsilon_{\mathrm{s}}\, \omega_{i,0} \tag{S4}$$

with the Grüneisen parameters $\gamma_{\mathrm{G}} = 1.8$ and $\gamma_{\mathrm{2D}} = 2.4$, as measured in similar circular graphene drums[11,15]. Eq. (S3) and (S4) are combined to estimate ξ. Supplementary Fig. **7** shows ε_{s} and $\Delta\omega_{\mathrm{2D}}$ as a function of ξ for $a = 3$ μm. By comparing to the experimental data recorded on device 1 (Supplementary Fig. **6** and Fig. 1d in the main text), we estimate $\xi \approx$ 42 nm and $\xi \approx$ 63 nm for $V_{\mathrm{dc}} = -6$ V and $V_{\mathrm{dc}} = -8$ V, respectively.

Starting from the estimated ξ based on the V_{dc}-dependent Raman mode frequencies, we can further cross-check our calibration by another method based on the dependence of Raman intensities (I_{G}, I_{2D}) on ξ (Supplementary Note 4 and Supplementary Fig. **5**c,d and **8**). The very good match between experimentally measured I_{G}, I_{2D}, their ratio ($I_{\mathrm{2D}}/I_{\mathrm{G}}$)

and calculations based on an optical interference model[10–12] allows us to further validate our calibration of ξ.

From Eq. (S3), the strain sensitivity can be obtained:

$$\frac{\partial \varepsilon_{\mathrm{s}}}{\partial \xi} = \frac{4\xi}{3a^2}. \tag{S5}$$

To obtain a larger sensitivity towards strain, dynamical Raman measurements were performed at sufficiently large V_{dc} to yield sizeable ξ, while at the same maintainting the graphene drum at reasonable distance ($\gtrsim$ 200 nm) from the Si/SiO_2 substrate and avoiding sample collapse and limiting electrostatic non-linearities[9].

Supplementary Note 4. Static displacement and equilibrium position shift

Determination of the static displacement

Using an multiple reflection model as in Supplementary Ref. 10–12, the intensity enhancement factors of the G- and 2D-mode features can be calculated as a function of ξ for $d_{\mathrm{SiO_2}} = 35$ nm, $d_{\mathrm{vac}} = 250$ nm, and a laser wavelength of 632.8 nm (Supplementary Fig. **5**c,d). Both I_{G} and I_{2D} monotonically increase with ξ for $\xi \lesssim 125$ nm, above which they monotonically decrease after reaching an intensity maximum. In particular, $I_{\mathrm{G}}(\xi)$ and $I_{\mathrm{2D}}(\xi)$ can be approximated as linear in the range $\xi = 20 - 70$ nm (Supplementary Fig. **8**), which corresponds to the static displacements explored in our study. The data points in Supplementary Fig. **8**a represent the equilibrium deflection (ξ) obtained at various V_{dc} and extracted from the measured Raman G- and 2D-mode frequencies (Supplementary Note 3 and Supplementary Fig. **6**-**7**). We can see that using an appropriate scaling factor that essentially accounts for the Raman susceptibilities of the G- and 2D-modes in the "interference-free" case[12,16], the intensity ratio $I_{\mathrm{2D}}/I_{\mathrm{G}}$ matches very well with theoretical predictions (Supplementary Fig. **8**b). This agreement validates our strain-based estimation of ξ and provides a solid ground to calibrate the RMS displacements (Supplementary Note 5).

Equilibrium position shift in the driven regime

In the linear regime, a graphene drum vibrates harmonically in a symmetric potential $U(\xi)$ (inset in Fig. 2e in the main text) with respect to the static equilibrium displacement ξ_{eq} (Fig. 2e). Under non-linear driving, the displacements are large enough such that the drum explores an asymmetric potential[17,18]. The drum now vibrates symmetrically with respect to an equilibrium position shifted by $\Delta\xi_{\mathrm{eq}}$, for which the Raman intensity enhancement factor (Supplementary Fig. **5**, **6**, **8**) is different. As a result, the measured Raman intensities become dependent on the driving force as the graphene drum is driven non-linearly, as evidenced in Fig. 2e and 3a, where Raman intensity drops by $\sim 20\%$ (at $V_{\mathrm{dc}} = -8$ V) and $\sim 10\%$ (at $V_{\mathrm{dc}} = -6$ V) are consistently observed. As discussed in the main text, these intensity drops correspond to an upshift of the equilibrium position (Supplementary Fig. **8**). Similar equilibrium position upshifts are discussed in device 3 (Fig. 4).

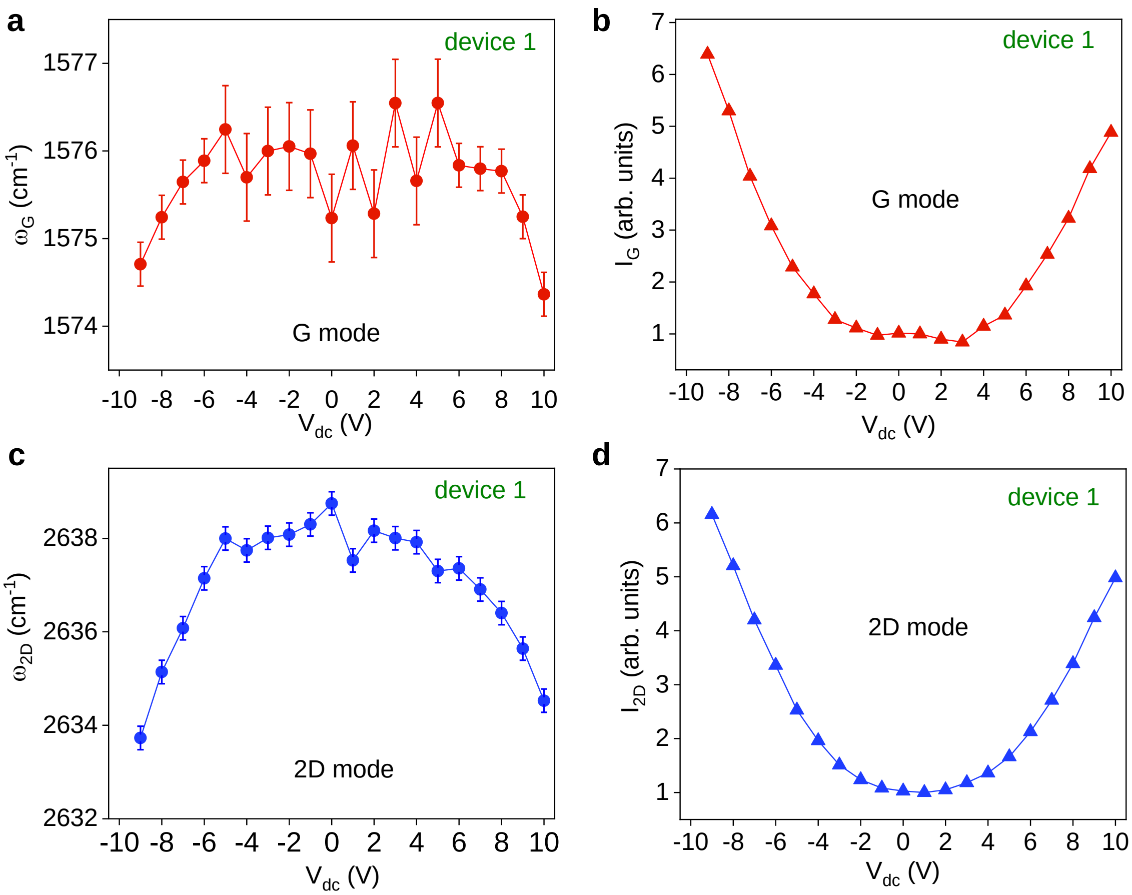

Supplementary Figure 6. **Probing static strain in an electrostatically gated graphene drum.** Frequency and integrated intensity of the G- (**a** and **b**, respectively) and 2D-mode (**c** and **d**, respectively) features as a function of V_{dc} in device 1 (see also Fig. 1d in the main manuscript for selected raw spectra). The integrated intensities are normalized with respect to the values measured at $V_{\mathrm{dc}} = 1$ V.

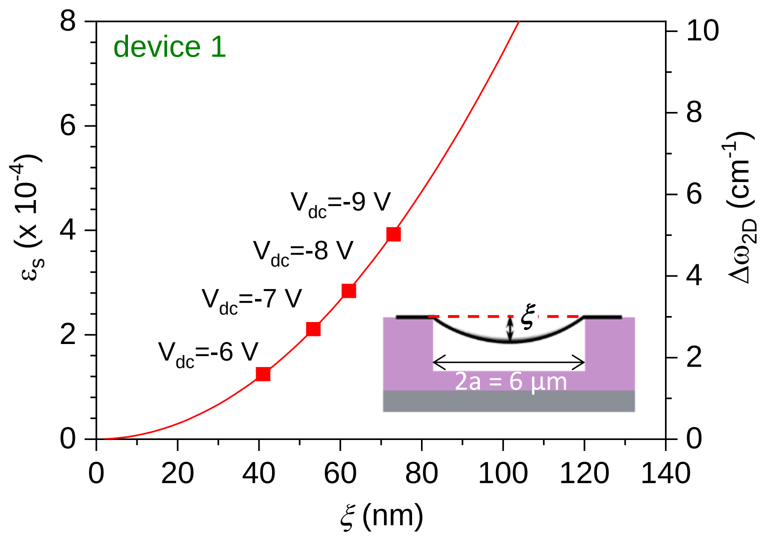

Supplementary Figure 7. **Determination of the static tensile strain and static deflection.** Calculated static strain ε_s using Eq. (S3) and corresponding frequency softening (Supplementary Note 3) of the 2D mode $\Delta\omega_{2D}$ as a function of ξ for a hole diameter $2a = 6\ \mu$m. The red symbols show $\Delta\omega_{2D}$ (from Supplementary Fig. **6**c) and the estimated ξ for V_{dc} ranging from -5 V to -8 V. The device geometry is recalled as an inset.

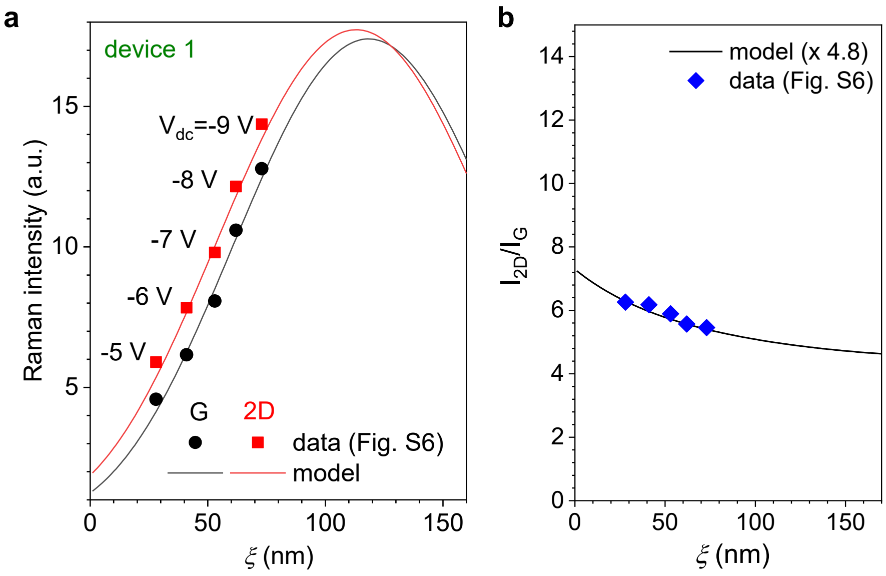

Supplementary Figure 8. Raman scattering intensity as a function of the static deflection. a, Calculated Raman intensity enhancement factors (solid lines) and measured Raman intensities (symbols) for the G (black) and 2D (red) modes as a function of ξ. The G- and 2D-mode intensities are extracted from Supplementary Fig. **6**b and d, respectively and scaled by a constant factor to allow comparison with the Raman intensity enhancement factors (Supplementary Fig. **5**c,d). The values of ξ associated with the experimental data are deduced from the strain-induced 2D-mode softening (Supplementary Note 4 and Supplementary Fig. **7**). **b,** Measured Raman intensity ratio (I_{2D}/I_G) as a function of ξ (blue symbols). The solid line is the ratio of the Raman intensity enhancement factors multiplied by a scaling factor of 4.8 that corresponds to the "interference-free case"[16].

Supplementary Note 5. Displacement calibration

Calibration Methods

A careful displacement calibration is essential to make sure that our assumption of a constant optomechanical transduction coefficient remains valid at the largest displacements attained in the non-linear regime. In addition, displacement calibration permits an estimation of the effective mass (see below) and allow demonstrating the pristine character of our samples and the generality of our findings.

The RMS displacements z_{rms} of our monolayer graphene drums are calibrated using three distinct methods described in the following subsections. The transduction coefficients β_i (nm/mV) (with $i = 1, 2, 3$) that connect the RMS voltage measured with our lock-in amplifier to z_{rms} are found to be very similar for the 3 methods and are summarized in Table **1** for device 2 at Vdc $= -8$ V.

Calibration method	β_i (nm/mV), $i = 1, 2, 3$
C_1 : Thermal noise	1.1 ± 0.15
C_2 : DC reflectance and Raman spectroscopy	1.0 ± 0.10
C_3 : DC reflectance and interference model	1.2 ± 0.20

Supplementary Table 1. **Diplacement calibration methods.** Transduction coefficient β_i (nm/mV), $i = 1, 2, 3$ connecting the measured RMS voltage on our lock-in amplifier to the measured RMS displacement z_{rms} of a driven graphene drum for three calibration methods (C_i, $i = 1, 2, 3$). Measurements were performed on device 2 at Vdc $= -8$ V.

C_1: Thermal noise

The mechanical oscillations of the graphene drum its thermal noise power spectral density (PSD) are related via[19]:

$$\langle z_n^2(t)\rangle = \int_0^\infty df\, S_{zz}(f) \tag{S6}$$

where $f = \Omega/2\pi$ is the mechanical frequency, $\langle z_n^2(t) \rangle$ is the mean-square amplitude of vibration of the n-th mode, which one-sided displacement spectral density $S_{zz}(f)$ writes:

$$S_{zz}(f) = \frac{k_B T f_n}{2\pi^3 \widetilde{m}_n Q_n \left[(f^2 - f_n^2)^2 + (f f_n/Q_n)^2\right]} \tag{S7}$$

where k_B, T, f_n, Q_n and $\widetilde{m}_n$ are the Boltzmann constant, the temperature (here taken equal to the ambient temperature), the resonance frequency, the quality factor and the effective mass of the n-th mode, respectively. Importantly, the surface mass density of our drum is assumed to be equal to that of pristine monolayer graphene (see below for a discussion on the relevance of this assumption). In the following, we will focus on the fundamental mechanical mode discussed in the main text.

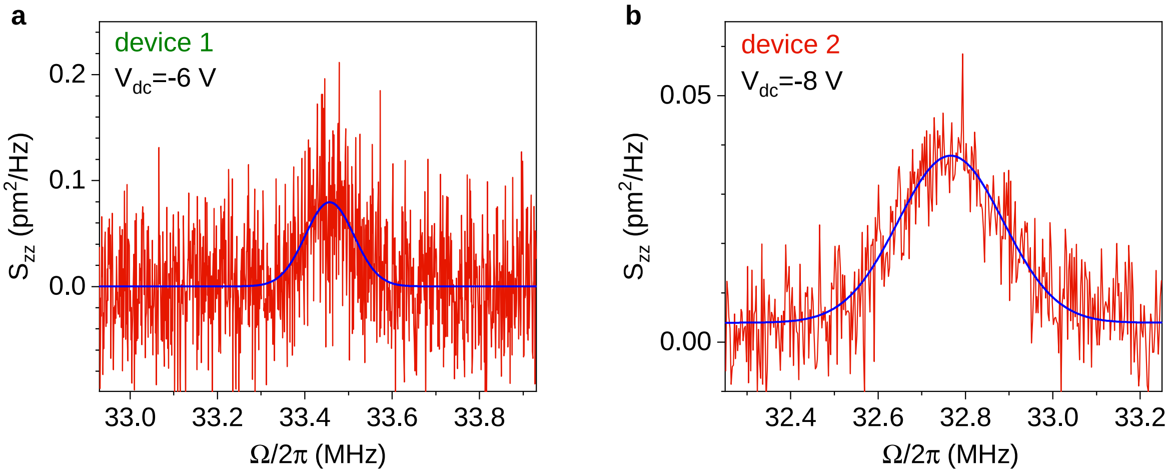

Supplementary Figure 9. Displacement calibration method C_1. Thermal noise power spectral density (PSD) of the fundamental mode of device 1 at $V_{dc} = -6$ V (**a**) and device 2 at $V_{dc} = -8$ V (**b**). The blue curves are the fit of the PSD using Eq. (S7).

The thermal noise PSD $S_{zz}(f)$ of the graphene drum is determined from the spectrum $V(f)$ of the output voltage of our avalanche photodiode, measured using a spectrum analyser. The resulting PSD is $S_{VV}(f) = V(f)^2/\Delta f$, where Δf is the resolution bandwidth (typically in the $10^2 - 10^3$ Hz range). The measured signal includes a flat noise floor (S_{VV}^w) due to the dark current noise of the photodiode and other sources of white noise and is connected to $S_{zz}(f)$ through:

$$S_{VV}(f) = S_{VV}^w + \eta S_{zz}(f), \tag{S8}$$

where η is another transduction coefficient expressed in V^2/m^2. η is obtained by fitting the measured $S_{VV}(f)$ by Eq. (S8), as in Supplementary Supplementary Fig. **9**. Finally, to calibrate the mechanical amplitude of the driven graphene drum measured using our lock-in amplifier, we simultaneously record the mechanical amplitude in the linear regime (typically with $V_{\mathrm{ac}} = 1$ mV) using the spectrum analyser and our lock-in amplifier and deduce β_1 (Table **1**). This calibration method was applied to all the devices studied in this work at various V_{dc}.

DC reflectance-based methods

The following two methods rely on a measurement of the DC reflectance of the sample (proportional to the intensity of the 632.8 nm laser beam reflected by the sample, see Supplementary Fig. **5**) as a function of V_{dc}, combined with a calibration of the gate-dependent static deflection ξ (Supplementary Fig. **5** and Supplementary Fig. **7**). Both methods connect the DC reflectance to ξ and yield the transduction coefficients β_2 and β_3.

C_2: DC reflectance and Raman spectroscopy. With calibration C_2, ξ is estimated through the gate-dependent spectral shifts of the Raman G and 2D modes as discussed in Supplementary Note 3, Supplementary Note 4 and Supplementary Fig. **6**-**7**). Coincidentally, the gate-induced changes of the DC reflectance are monitored with our lock-in amplifier.

C_3: DC reflectance and interference model. As discussed in Supplementary Note 2 and Supplementary Fig. **5**, an interference calculation[8,10] can be applied to obtain the reflectance of our samples as a function of ξ. Supplementary Fig. **10** shows the calculated reflectance together with our measurements of the reflected laser intensity vs V_{dc}, scaled to match the simulated values.

Discussion on the effective mass of graphene drums

The calibration of the displacement of a nanomechanical system with thermal noise measurements (C_1) requires accurate knowledge of its effective mass. Here, we have considered the surface mass density of pristine monolayer graphene ($\approx 7.5 \times 10^{-7}$ kg/m^2). For the fundamental mechanical mode of circular drum, the rest mass of graphene has to be scaled by a

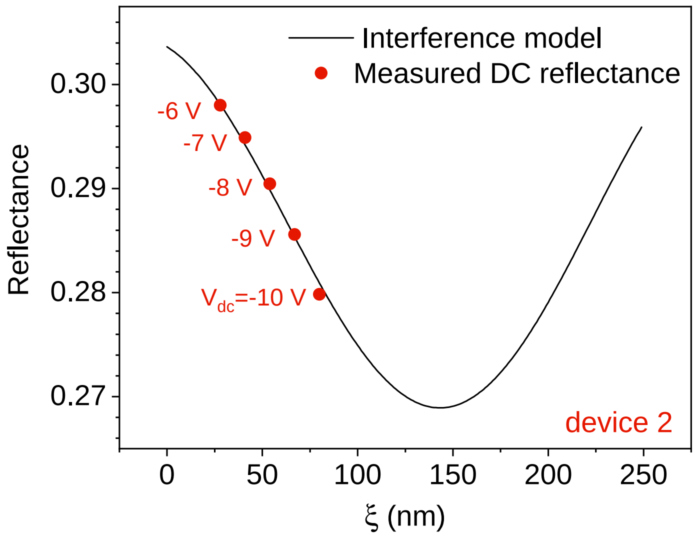

Supplementary Figure 10. **Displacement calibration method** C_3**.** Calculated (solid line) and measured (symbols) sample reflectance at 632.8 nm as a function of the static displacement ξ.

factor ≈ 0.27 (Supplementary Ref. 19), such that the *effective* mass of our 6 μm−diameter drum is $\widetilde{m}_0 \approx 5.7 \times 10^{-18}$ kg. Calibration C_2 and C_3 are totally independent of $\widetilde{m}$ and yield transduction coefficients $\beta_{2,3}$ that are, within experimental accuracy, equal the coefficient β_1 obtained using thermal noise measurements considering $\widetilde{m}_0$ (see values and associated errorbars in Table **1**). This key result justifies our assumption that $\widetilde{m} = \widetilde{m}_0$.

Following previous reports, we could have expected that $\widetilde{m}$ would *a priori* exceed $\widetilde{m}_0$ due to the presence of molecular adsorbates and other sources of contamination[20]. In addition, graphene drums and blisters, in particular when made from wet-transfer of graphene layers grown by chemical vapor deposition (CVD), are known to exhibit rippling and crumpling[21]. The resulting hidden area effects lead to discrepancies between the levels of stain determined through Raman and interferometric measurements[22] and thus affect our displacement and strain calibration. Here, the excellent agreement between calibration methods C_2 and C_3 demonstrates that our graphene drums are immune from hidden area effects, as previously observed in our blister test on pristine suspended graphene, where a Young's modulus matching that of bulk graphite was found[11].

Our devices are made from freshly exfoliated natural graphite flakes using a dry, resist-free transfer method and then held in high vacuum. Such freely suspended graphene membranes have consistently shown intrinsic electronic[23] and optical[6,24,25] properties. Our study also

demonstrates that the same holds for their mechanical figures of merit.

Let us note in closing that assuming $\widetilde{m} > \widetilde{m}_0$ when using method C_1 would lead to smaller calibrated displacements than those estimated assuming $\widetilde{m}_0$. Smaller displacements would lead to smaller values of $\varepsilon_{\mathrm{d}}^{\mathrm{h}}$ calculated through Eq. (S18) and to a larger discrepancy between $\varepsilon_{\mathrm{d}}^{\mathrm{h}}$ and the enhanced ε_{d} determined from our Raman measurements in resonantly driven graphene drums.

Supplementary Note 6. Mechanical response of driven graphene drums

The displacement of our graphene drums can be modeled as that of a driven non-linear oscillator by[17]:

$$\ddot{z} + \frac{\Omega_0}{Q}\dot{z} + \Omega_0^2 z + \alpha_2 z^2 + \alpha_3 z^3 = \frac{\widetilde{F}_{el}}{\widetilde{m}}\cos(\Omega t) \tag{S9}$$

where z is the mechanical displacement at the membrane center, $\Omega_0/2\pi$ is the resonance frequency in the linear regime, Q is the quality factor and Ω_0/Q is the linear damping rate, α_2, α_3 are the quadratic and the cubic spring constant, respectively. Finally, $\widetilde{m} = 0.27\, m_0$ (with m_0 the rest mass of the graphene drum) is the effective mass with a correction factor that accounts for the mode shape of the fundamental resonance of a clamped circular membrane[9,19,20,26] and $\widetilde{F}_{el}$ is the effective applied electrostatic force.[27]

Linear response

In the linear response regime, $\alpha_{2,3} = 0$, Eq. (S9) is the well-known differential equation of a driven harmonic oscillator. Assuming a harmonic solution $z(t) = z_0 e^{i\Omega t}$, one gets:

$$z_0 = \frac{\widetilde{F}_{\mathrm{el}}/\widetilde{m}}{\Omega_0^2 - \Omega^2 + \mathrm{i}\,\Omega_0\Omega/\mathrm{Q}}. \tag{S10}$$

For the fundamental mechanical mode of a thin circular membrane resonator under a sufficiently high built-in tension T_0 (as is the case for our graphene drums) Ω_0 writes[28]

$$\Omega_0 = 2\pi f_0 = u_{01}\sqrt{\frac{T_0}{\rho_{1\mathrm{LG}}\, a^2}}, \tag{S11}$$

where $\rho_{1\mathrm{LG}} \approx 7.5\times10^{-7}\ \mathrm{kg/m^2}$ is the surface mass density of pristine graphene and $u_{01} \approx 2.405$ is the first zero of the zero-order Bessel function. Therefore, T_0 writes

$$T_0 = 0.69\,\pi^2 f_0^2\,\rho_{1\mathrm{LG}}\,a^2, \tag{S12}$$

where $T_0 = E_{1\mathrm{LG}}\ \varepsilon_{\mathrm{s}}/\,(1-\nu)$ (Supplementary Ref. 28), with $E_{1\mathrm{LG}} = 340\ \mathrm{Nm^{-1}}$ and $\nu = 0.16$ the Young's modulus and Poisson ratio of pristine monolayer graphene, respectively[29]. Eq. (S12) is then used to compute the built-in and the gate-induced static strain discussed in the text. These strain values can be compared with estimates from the G- and 2D-mode softenings.

From Eq. (S10), we get

$$|z_0|^2 = \frac{\left(\widetilde{F}_{\rm el}/\widetilde{m}\right)^2}{(\Omega_0^2 - \Omega^2)^2 + (\Omega\Omega_0/Q)^2}, \tag{S13}$$

Eq. (S13) can be used to fit the frequency-response curve in the linear response region and extract Q, as in Fig. 1. Furthermore, near resonance ($|\Omega - \Omega_0| \ll \Omega_0$) and for $Q \gg 1$, Eq. (S13) simplifies as

$$|z_0|^2 = \frac{\widetilde{F}_{el}^2}{4\widetilde{m}^2\Omega_0^2} \frac{1}{(\Omega - \Omega_0)^2 + \Omega_0^2/4Q^2}, \tag{S14}$$

which is a Lorentzian lineshape with full width at half maximum (FWHM) Ω_0/Q.

Non-linear response

Eq. (S9) can be rewritten by introducing an effective cubic spring constant[17,20] given by

$$\widetilde{\alpha}_3 = \alpha_3 - \frac{10\alpha_2^2}{9\Omega_0^2}, \tag{S15}$$

such that a Duffing-like equation can still be written as

$$\ddot{z} + \frac{\Omega_0}{Q}\dot{z} + \Omega_0^2 z + \widetilde{\alpha}_3 z^3 = \frac{\widetilde{F}_{el}}{\widetilde{m}}\cos(\Omega t). \tag{S16}$$

To obtain $\widetilde{\alpha}_3$, we can approximate the solution of Eq. S16 by a truncated Fourier series, restricted here to first order. This approach allows establishing the analytical expression of the so-called *backbone curve* that connects the maximum amplitude z_0 to the drive frequency $\widetilde{\Omega}_0/2\pi$ at which it is obtained. Following Refs. 9 and 17, we get

$$\widetilde{\Omega}_0 = \Omega_0 + \frac{3}{8}\frac{\widetilde{\alpha}_3}{\Omega_0} z_0^2. \tag{S17}$$

As the driving force is increased, the onset of third-order non-linearities leads to resonance frequency hardening for $\widetilde{\alpha}_3 > 0$ (see data at $V_{\rm dc} = -6$ V in Fig. 3 and Supplementary Fig. **11** and at $V_{\rm dc} = -5$ V in Supplementary Fig. **12**), and to resonance frequency softening for $\widetilde{\alpha}_3 < 0$ (see Fig. 2 in the main text, for $V_{\rm dc} = -8$ V), respectively. As expected from Eq. (S17), a parabolic backbone curve is observed at $V_{\rm dc} = -8$ V and to a lesser extent at $V_{\rm dc} = -5$ V (Supplementary Fig. **12**). However, the backbone curve fully saturates at $V_{\rm dc} = -6$ V for $V_{\rm ac} > 40$ mV (Fig. 3a, Fig. 3d and Supplementary Fig. **12**). In this

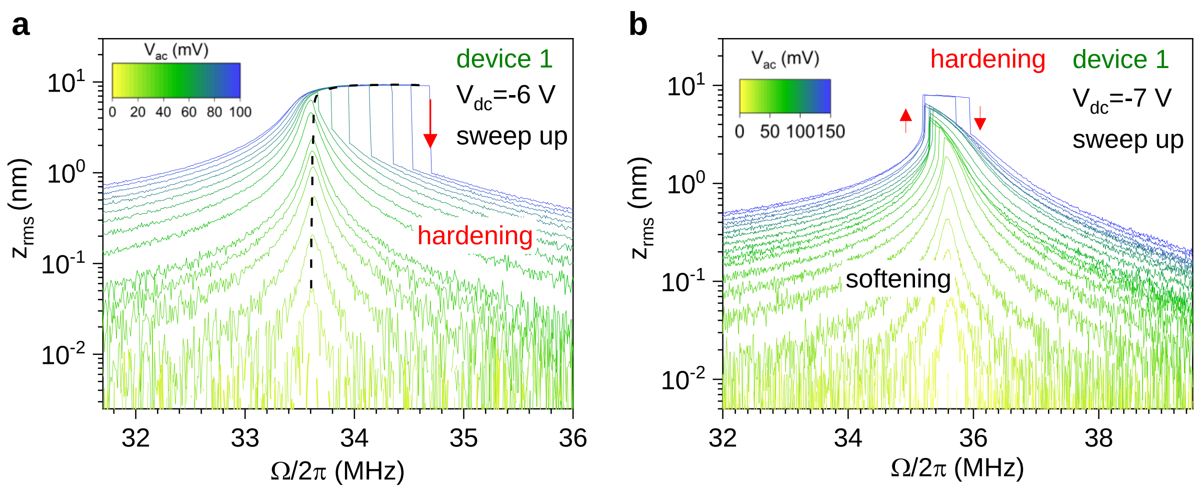

Supplementary Figure 11. **Mechanical non-linearities in graphene drums (1).** Frequency-response curves obtained by sweeping the drive frequency upward at $V_{dc} = -6$ V (**a**) and $V_{dc} = -7$ V (**b**) on device 1. At $V_{dc} = -7$ V, a nonlinear softening to hardening transition is revealed above $V_{\rm ac} = 130$ mV.

strongly non-linear regime, sizeable Fourier components are expected at harmonics of the drive frequency, as experimentally verified on device 2 in Supplementary Fig. **17**, and the first order expansion is insufficient. Non-linearities can be either be i) intrinsic to graphene, e.g. due to its cubic spring constant[29] but also ii) electrostatically-induced by the dependence of the gate capacitance on the distance between the vibrating graphene drum and the Si backgate[9] or iii) geometrically induced by the displacement-dependent tension induced by the vibrations of the drum[30]. For instance, using Eq. (12) and (26) in the supplementary information of Supplementary Ref. 9, we can estimate that the ratio between the third order intrinsic stiffness of graphene and the gate-induced third order softening term is close to 3 at $V_{\rm dc} = -6$ V and near unity at $V_{\rm dc} = -8$ V. At the same time, we estimate that the gate-induced second order spring constant (α_2) is large enough such that Eq. (S15) yields $\widetilde{\alpha}_3 \approx -\frac{10\alpha_2^2}{9\Omega_0^2} \approx -1 \times 10^{32}$ m^{2}s^{-2} at $V_{\rm dc} = -8$ V. This value is in good agreement with the experimental value extracted from a fit of the backbone curve in Supplementary Fig. **12**a. At this point, geometrical non-linearities have not been considered and are discussed below.

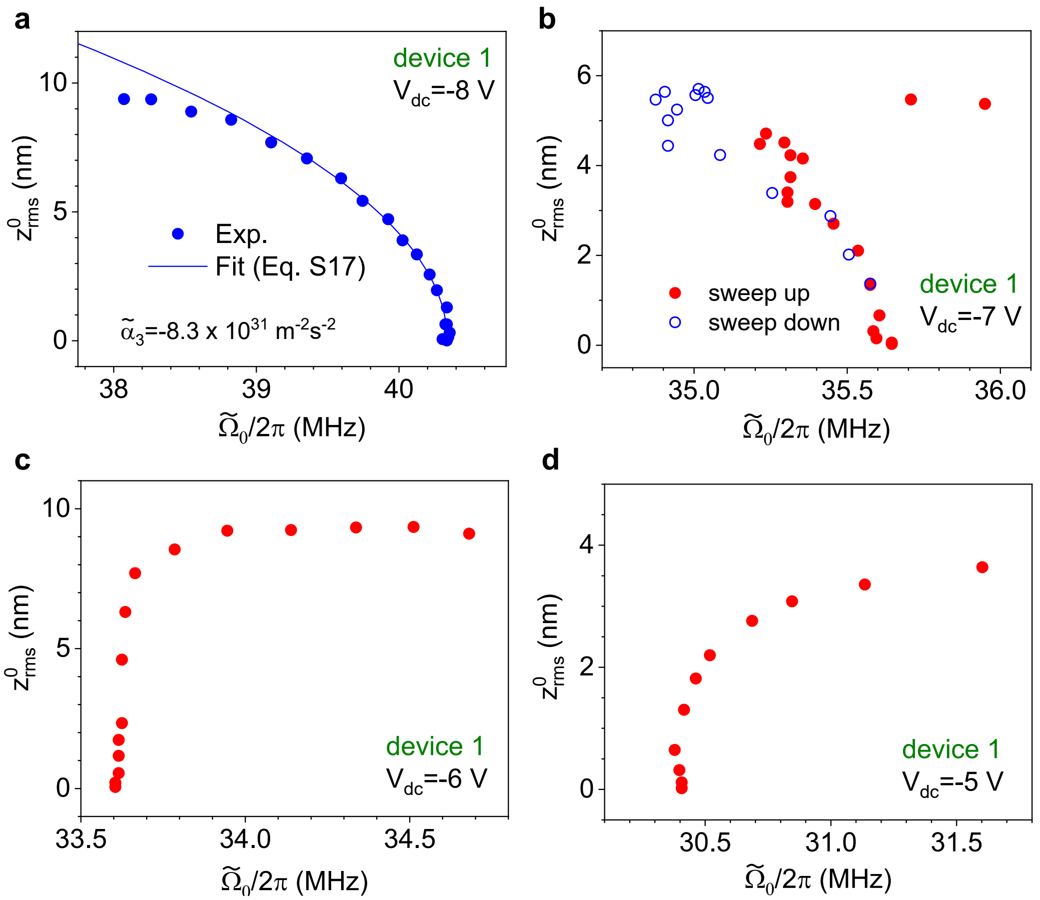

Supplementary Figure 12. **Mechanical non-linearities in graphene drums (2).** Backbone curves (see Supplementary Note 6) recorded on device 1 at four distinct gate biases ($V_{dc} = -8, -7, -6, -5$ V in **a**, **b**, **c**, **d**, respectively) . At $V_{dc} = -8$ V, a fit using Eq. (S17) allows to extract the non-linear coefficient $\tilde{\alpha}_3$ in Eq. (S16). The data at $V_{dc} = -8$ V and $V_{dc} = -6$ V are plotted in Fig. 2f and 3d, respectively.

Supplementary Note 7. Dynamical strain and non-linearities

In this Supplementary Note, we provide insights into the origin of the enhanced dynamical strain observed in our experiments.

Dynamical strain induced by harmonic vibrations

Let us return to the simple one-dimensional model introduced in Supplementary Note 3. We first consider a given RMS amplitude z_{rms} and compare the values of dynamically-induced strain ε_{d} measured under strong non-linear driving to the values expected with harmonic oscillations. For simplicity, we assume that under the application of a sinusoidal driving force at frequency $\Omega/2\pi$, the drum maintains a parabolic mode shape and that the time-dependent displacement at the membrane center writes $\xi(t) = \xi + \sqrt{2}\, z_{\mathrm{rms}} \cos(\Omega t + \varphi)$, with φ the phase difference between the drive and the mechanical response (Supplementary Note 6). The time-averaged *harmonic* dynamical strain $\varepsilon_{\mathrm{d}}^{\mathrm{h}}$ can be estimated by inserting $\xi(t)$ into Eq. (S3) and averaging over one oscillation period. Since the crossed term $2\sqrt{2}\,\xi z_{\mathrm{rms}} \cos(\Omega t+\varphi)$ averages out to zero, we obtain

$$\varepsilon_{\mathrm{d}}^{\mathrm{h}} = \frac{2}{3}\left(\frac{z_{\mathrm{rms}}}{a}\right)^2 . \tag{S18}$$

Eq. (S18) is then used with the measured RMS displacements z_{rms} to compare $\varepsilon_{\mathrm{d}}^{\mathrm{h}}$ with the measured ε_{d} in Fig. 2-4 in the main manuscript. With $z_{\mathrm{rms}} = 9$ nm and $a = 3$ μm, Eq. (S18) yields $\varepsilon_{\mathrm{d}}^{\mathrm{h}} = 6 \times 10^{-6}$, a value that is about 40 times smaller than the measured ε_{d} obtained when z_{rms} reaches 9 nm (Fig. 2f). This obvious discrepancy suggests that non-linearities result in anharmonic oscillations and complex mode profiles, leading to enhanced ε_{d}, as further discussed below.

Geometrical non-linearities

We now provide additional insights into the key observation in Fig. 2f and 3d that the non-linear frequency shift $\delta = \frac{\widetilde{\Omega}_0-\Omega_0}{\Omega_0}$ (Eq. (S17)) is proportional to the dynamical strain ε_{d}.

For the sake of simplicity, the static displacement profile introduced above will not be explicitly considered in the following discussion. For a given transverse vibrational mode (whose mode index n will be omitted in the following), the time and space-dependent dis-

placement of the resonator writes $u(x,t) = z(t)\phi(x)$, with $\phi(x)$ the dimensionless mode profile (defined such that $\phi(0) \equiv 1$) and $z(t)$ the displacement introduced in Eq. (S9). With the reasonable assumption that $|\phi'(x)|\, a \ll 1$, the time-averaged longitudinal dynamical strain writes

$$\varepsilon_{\rm d} = \frac{z_{\rm rms}^2}{4a} \int_{-a}^{a} \left[\phi'(x)\right]^2 dx. \tag{S19}$$

We will restrict ourselves to the simple case of a third order geometrical non-linearity, and consider a Duffing-like equation (i.e., Eq. (S9) with $\alpha_2 = 0$ and $\alpha_3 \neq 0$). The effective mass, the linear and non-linear spring constants associated with the mechanical mode under study can be written, respectively as[30]

$$\widetilde{m} = \frac{m_0}{2a} \int_{-a}^{a} \phi_n^2(x) dx \tag{S20a}$$

$$k_1 = \widetilde{m}\Omega_0^2 = \sigma A \int_{-a}^{a} \left[\phi'(x)\right]^2 dx \tag{S20b}$$

$$k_3 = \widetilde{m}\alpha_3 = \frac{EA}{4a} \left(\int_{-a}^{a} \left[\phi'(x)\right]^2 dx \right)^2 \tag{S20c}$$

where σ and E are the initial stress and bulk Young's modulus. Eq. (S17) can be recast as

$$\delta = \frac{3}{8}\frac{k_3}{k_1} z_0^2 = \frac{3}{32}\frac{z_0^2}{a}\frac{E}{\sigma} \int_{-a}^{a} \left[\phi'(x)\right]^2 dx. \tag{S21}$$

Using Eq. (S21) and (S19), and assuming that $z_{\rm rms}^2 \approx z_0^2/2$, we obtain

$$\delta \approx \frac{3}{4}\frac{E}{\sigma}\varepsilon_{\rm d}. \tag{S22}$$

Eq. (S22) thus establishes the proportionality between δ and $\varepsilon_{\rm d}$, in qualitative agreement with the results in Fig. 2f and Fig. 3d. As indicated in the main manuscript and in Supplementary Fig. **7**, for the values of $V_{\rm dc}$ used in our study (see also Fig. 2 and 3), the gate-induced static strain $\varepsilon_{\rm s} \approx \sigma/E \approx 2 \times 10^{-4}$ is close to these values of $\varepsilon_{\rm d}$ attained as $z_{\rm rms}$ saturates (Fig. 2f and 3d). With these values, Eq. (S22) would yield $\delta \sim 1$, in obvious contradiction with Fig. 2f, 3d, and **12** that show that $|\delta|$ does hardly exceed 5%. To explain this discrepancy, one should keep in mind that Eq. (S22) has been derived using solely third order geometrical non-linearities (Eq. (S20c)) to describe the Duffing coefficient and hence ignoring other intrinsic and electrostatically-induced non-linearities as discussed in Supplementary Note 6. These various non-lineartites lead to amplitude saturation and may cause the emergence of non-trivial mode profiles, with large gradients ($\phi'(x)$), as recently observed

experimentally[31]. From Eq. (S19), it is clear that sharp changes in the mode profiles will enhance ε_{d}. At the same time, non-linearities may lead to mechanical mode hardening (as in the case of a geometrical Duffing non-linearity described by Eq. (S20c)) or softening, as exemplified in Fig. 2 and discussed above (Eq. (S15), see also Supplementary Fig. **11** and Supplementary Fig. **12**). All in all, the measured values of δ result from the interplay between several sources of non-linearity listed above[9,30,32]. One may thus observe $|\delta|$ of a few % together with non-linearly enhanced ε_{d} that gets as large as ε_{s}. We conclude that our results strongly suggest that $\phi'(x)$ takes on large values on length scales that are significantly smaller than a that cannot be resolved using our diffraction-limited setup (see main text for details).

Supplementary Note 8. Effect of laser-induced heating

In our measurements, the laser spot is typically around 1.2 μm in diameter[12] and the laser power was set to $P_{\text{laser}} \sim$ 500 μW for the measurements in Fig. 1-3 and $P_{\text{laser}} \sim$ 200 μW for the measurements in Fig. 4. These values corresponds to a reasonable trade off to obtain a sufficiently large Raman signal without being perturbed by softening of the Raman modes due to laser-induced heating[33]. However, the photon flux on the suspended drum is sufficient to induce photothermal effects on its mechanical susceptibility[34]. As shown in Supplementary Fig. **13**, at $V_{\text{dc}} = -6$ V the resonance frequency $\Omega_0/2\pi \approx 30.6$ MHz is nearly independent on the laser power below a threshold $P_{\text{laser}} \approx 200$ μW, above which a linear increase in Ω_0 is found, as in previous reports[34]. To estimate the temperature (T) increase caused by laser heating, we extracted the thermally induced strain ε_T from the experimental data in Supplementary Fig. **13**a using Eq. (S11)

$$\varepsilon_T = \frac{1-\nu}{E_{1\text{LG}}} \times 0.69\,\pi^2 f(T)^2\,\rho_{1\text{LG}}\,a^2. \tag{S23}$$

As shown in Supplementary Fig. **13**b, above $P_{\text{laser}} \sim 200$ μW, the obtained values of ε_T increase linearly with P_{laser}. Using a thermal expansion coefficient $\kappa_T \approx -8 \times 10^{-6}$ K^{-1} (Supplementary Ref. 35), we estimate a temperature increase $\Delta T = -\varepsilon_T/\kappa_T \approx 2.5$ K at $P_{\text{laser}} = 500$ μW, a value that is about two orders of magnitude too small to account for the dynamical Raman frequency softenings discussed in the main text.

To further rule out laser-induced Raman frequency softening, we repeated the Raman measurements in driven graphene drums at $P_{\text{laser}} = 200$ μW, a value that is low enough to neglect photothermal effects on the mechanical resonance frequency (Supplementary Fig. **13**a,c). Supplementary Fig. **13**d shows the Raman 2D-mode spectra recorded under $V_{\text{dc}} = -6$V and $V_{\text{ac}} = 125$ mV at near-resonant and off-resonant drive frequencies. Raman frequency softening under resonant driving akin to Fig. 3 of the main text is clearly observed.

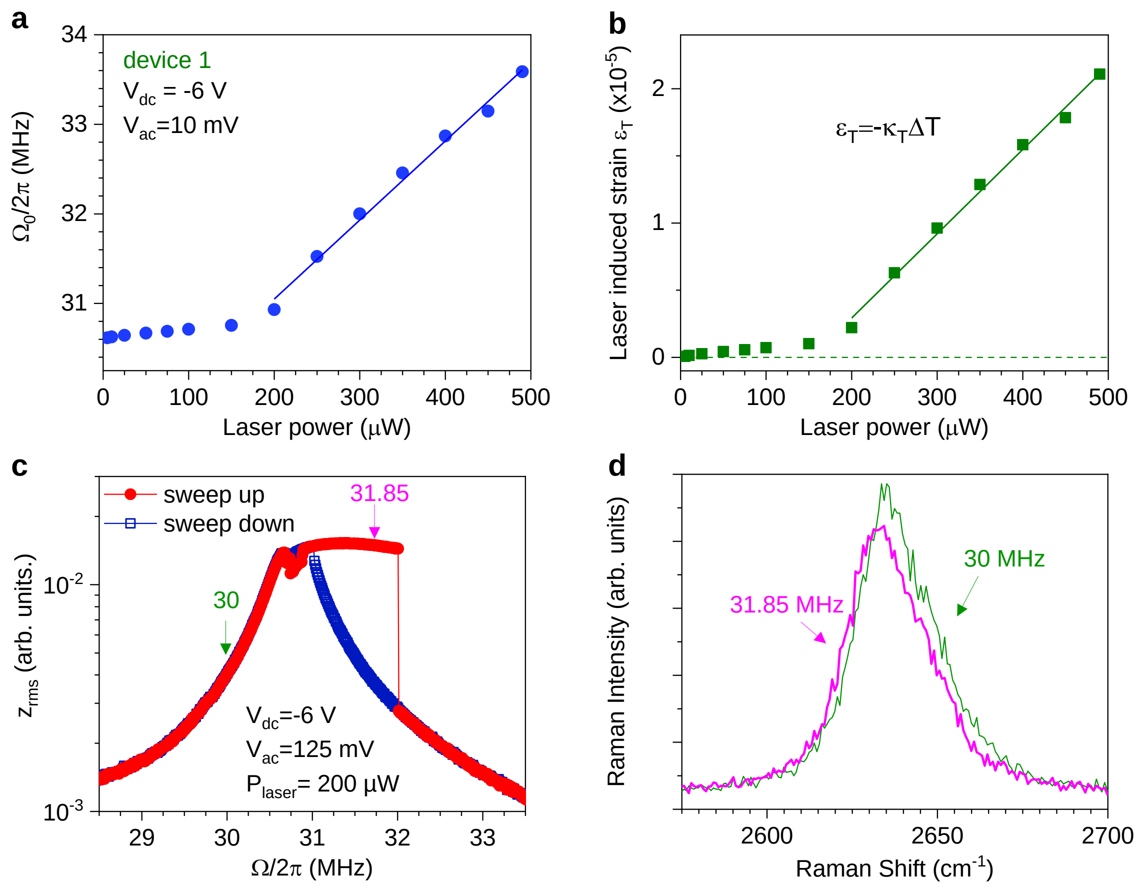

Supplementary Figure 13. Effects of laser-induced heating on the mechanical response of graphene drums. a, Resonance frequency $\Omega_0/2\pi$ measured as a function of the laser power in device 1. The blue line is a linear fit. **b,** Extracted stain (ε_T) as a function of laser power. The solid line is a linear fit (Supplementary Note 8). **c,** Frequency-dependent RMS displacement $z_{\rm rms}$ at $V_{\rm dc} = -6$ V and $V_{ac} = 150$ mV using a laser power 200 μW. **d,** Dynamical Raman spectra recorded in the aforementioned conditions under two distinct drive frequencies indicated by the green and pink arrows in (**c**).

Supplementary Note 9. Supplementary data on device 1

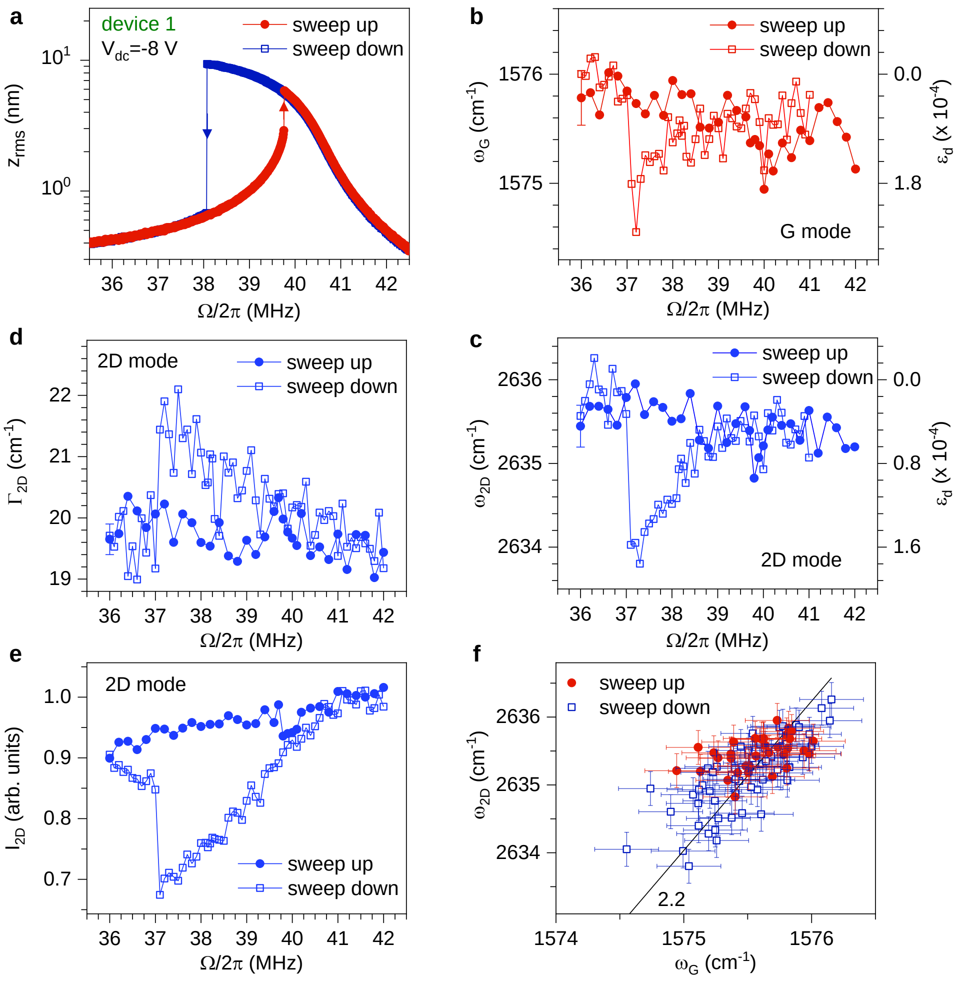

Supplementary Figure 14. Frequency-dependent dynamically-induced strain at V_{dc} = −8 V in device 1. a, Frequency-response curves on device 1 at V_{dc}=-8 V and V_{ac} =150 mV. The arrows denote the jump-up and jump-down frequencies. Frequencies of the Raman G mode (**b**) and 2D mode (**c**) as a function of $\Omega/2\pi$. FWHM (**d**) and integrated Raman intensity (**e**) of the 2D-mode feature as a function of $\Omega/2\pi$. **f,** Correlation between G- and 2D-mode frequencies. A straight black line with slope of 2.2 is a guide to the eye showing the expected correlation in the case of strain-induced phonon softening[11]. Only one error bar is included in (**b,d,e**) for clarity. The jump frequencies appear at drive frequencies that are slightly redshifted by ∼ 1 MHz relative to the frequency-response curves in (**a**). This effect is attributed to photothermally induced mechanical frequency downshift (Supplementary Note 8 and Supplementary Fig. **13**).

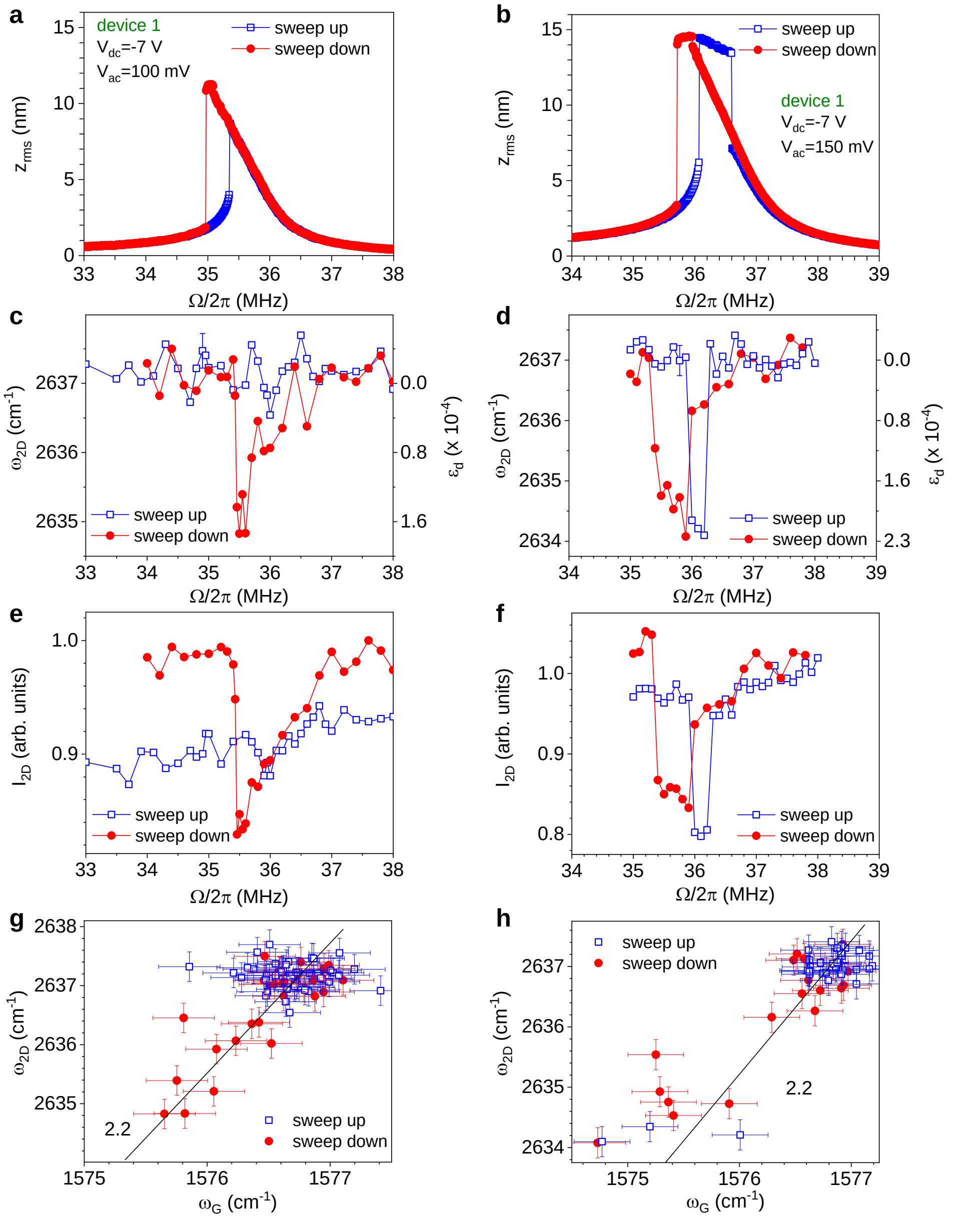

Supplementary Figure 15. Frequency-dependent dynamically-induced strain at $V_{dc} = -7$ V in device 1. Frequency-response curves measured on device 1 at $V_{dc} = -7$ V with $V_{ac} = 100$ mV (**a**) and $V_{ac} = 150$ mV (**b**). Raman 2D mode frequency ω_{2D} as a function of $\Omega/2\pi$ under $V_{ac} = 100$ mV (**c**) and $V_{ac} = 150$ mV (**d**), respectively. Corresponding integrated intensity I_{2D} (**e**, **f**) and correlations between the G- and 2D-mode frequencies **g, h**. The straight black line with a slope of 2.2 is a guide to the eye showing the expected correlation for strain-induced phonon softening. The jump frequencies appear at drive frequencies that are slightly shifted by $\lesssim 1$ MHz relative to the frequency-response curves in (**a**,**b**). This effect is attributed to photothermally induced mechanical frequency downshift (Supplementary Note 8 and Supplementary Fig. **13**).

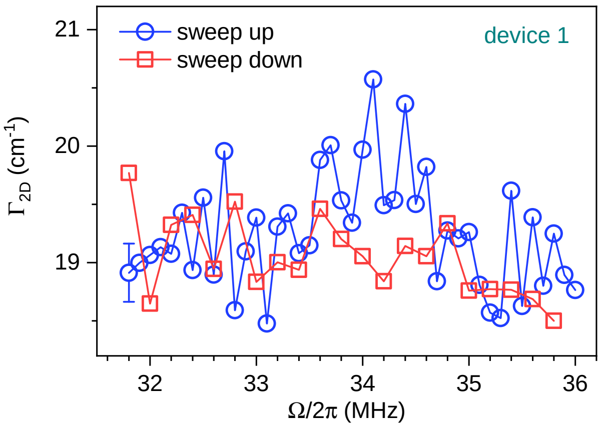

Supplementary Figure 16. Dynamically-induced 2D-mode broadening at $V_{\mathrm{dc}} = -6$ V in device 1. Full width at half maximum of the 2D mode feature $\gamma_{2\mathrm{D}}$ (we considered the $2\mathrm{D}^-$ component, see Supplementary Note 1) as a function of the drive frequency. This data is extracted from the measurements show in Fig. 3 of the main manuscript. A slight broadening is observable as the RMS amplitude saturates near $\Omega/2\pi = 34$ MHz (see Fig. 3a).

Supplementary Note 10. Supplementary data on device 2

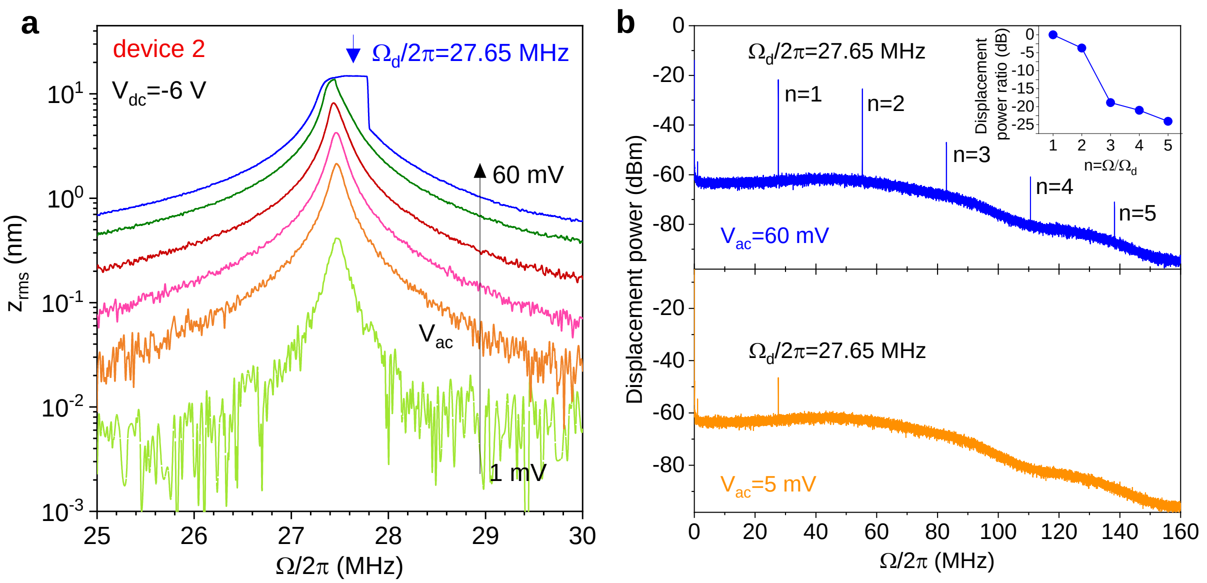

Supplementary Figure 17. **Harmonic generation under non-linear mechanical driving.** **a**, Frequency-response curve measured at $V_{\rm dc} = -6$ V with $V_{\rm ac}$ ranging from 5 mV up to 60 mV in device 2, a graphene drum similar to devices 1 and 2. The blue arrow denotes the drive frequency $\Omega_{\rm d}/2\pi$ used in (**b**). **b**, Broadband displacement power spectral density under $\Omega_{\rm d}/2\pi =$ 27.65 MHz. Bottom panel, with $V_{\rm ac} = 5$ mV; top panel, with $V_{\rm ac} = 60$ mV. Sizeable high-order harmonic components (here up to 5 $\Omega_{\rm d}/2\pi$) are revealed when the drum is resonantly driven with large amplitude. The 50 MHz bandwidth of our avalanche photodiode is clearly visible. Inset: Displacement power of the harmonics relative to the displacement power at $\Omega_{\rm d}$.

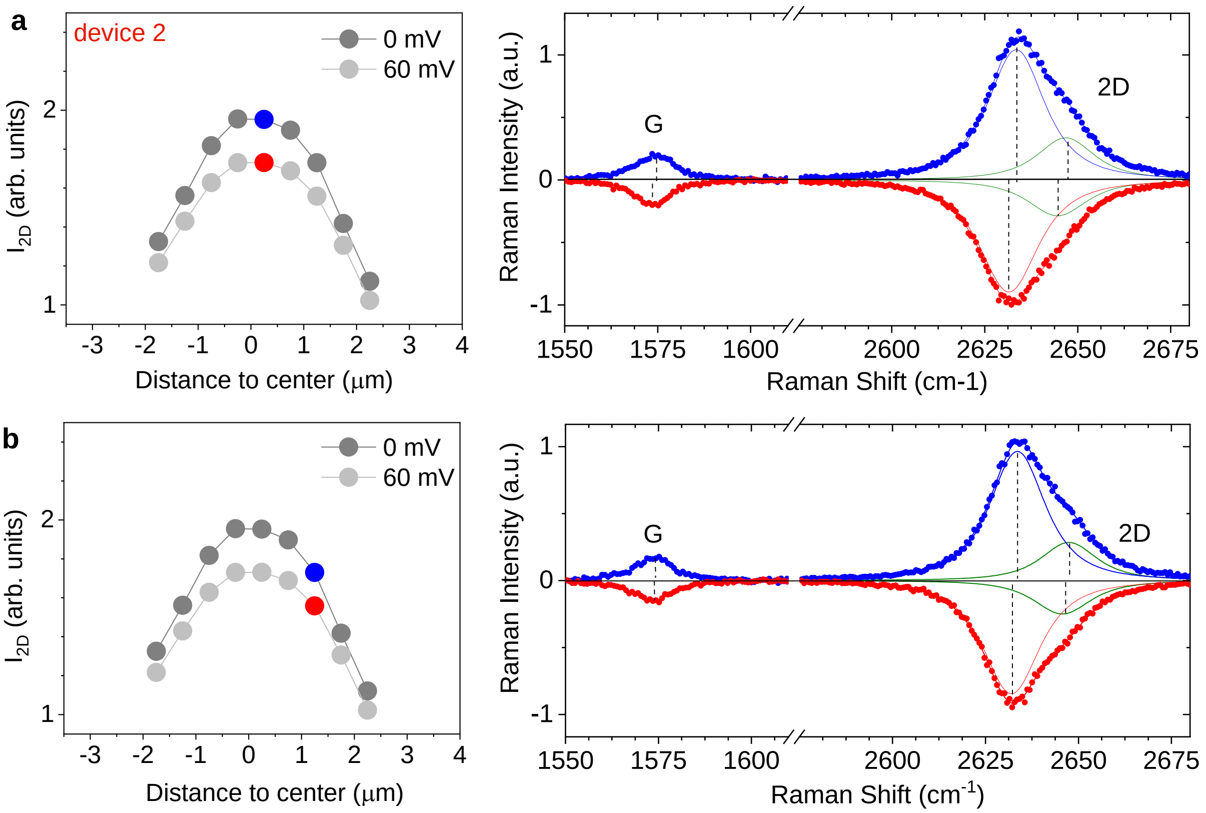

Supplementary Figure 18. **Spatially-revolved Raman spectroscopy in device 2.** Selected Raman spectra taken at the centre of device 2 (**a**) and 2 μm away from the centre (**b**) under $V_{\mathrm{dc}} = -6$ V and $V_{\mathrm{ac}} = 0$ (data in blue) and $V_{\mathrm{dc}} = -6$ V and $V_{\mathrm{ac}} = 60$ mV (data in red). See also Fig. 4 in the main text and related discussion.

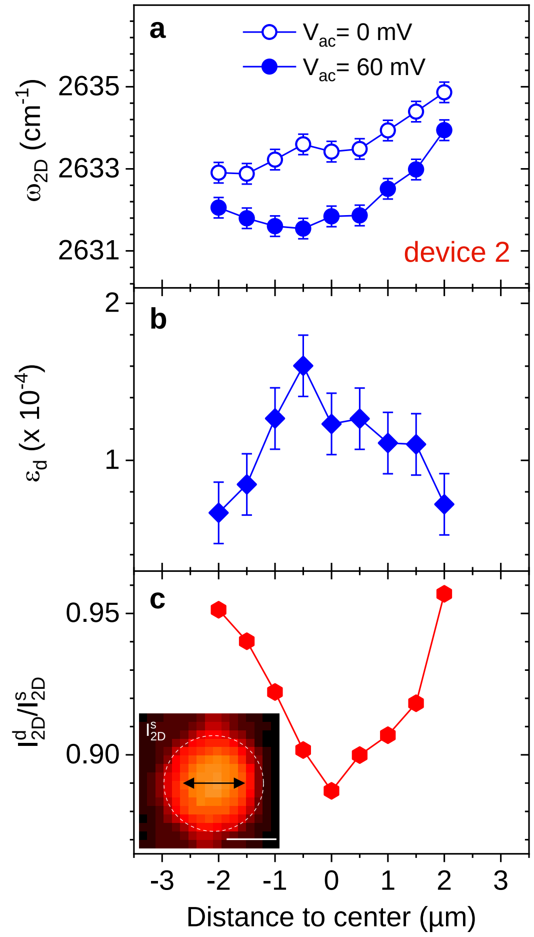

Supplementary Figure 19. **Spatially-revolved dynamically-induced strain in device 2.** **a**, Frequency of the Raman 2D mode along the cross-sections highlighted in **c** in a graphene drum (device 2, radius 3 μm) at $V_{\rm dc} = -6$ V and $V_{\rm ac} = 0$ mV (open symbols) and $V_{\rm ac} = 60$ mV (full symbols). **b**, Dynamical strain $\varepsilon_{\rm d}$ obtained from the difference of the data in **a**. **c**, Ratio of the Raman 2D-mode intensity in the driven ($I^{\rm d}_{\rm 2D}$) and static ($I^{\rm s}_{\rm 2D}$) cases. Inset: Map of the Raman 2D-mode intensity $I^{\rm s}_{\rm 2D}$ recorded on the graphene drum (see white dashed contour), at $V_{\rm dc} = -6$ V and $V_{\rm ac} = 0$ V. The double arrow indicates the location of the line scan. The scale bar is 3 μm. See also Figure 4 in the main text and related discussion.

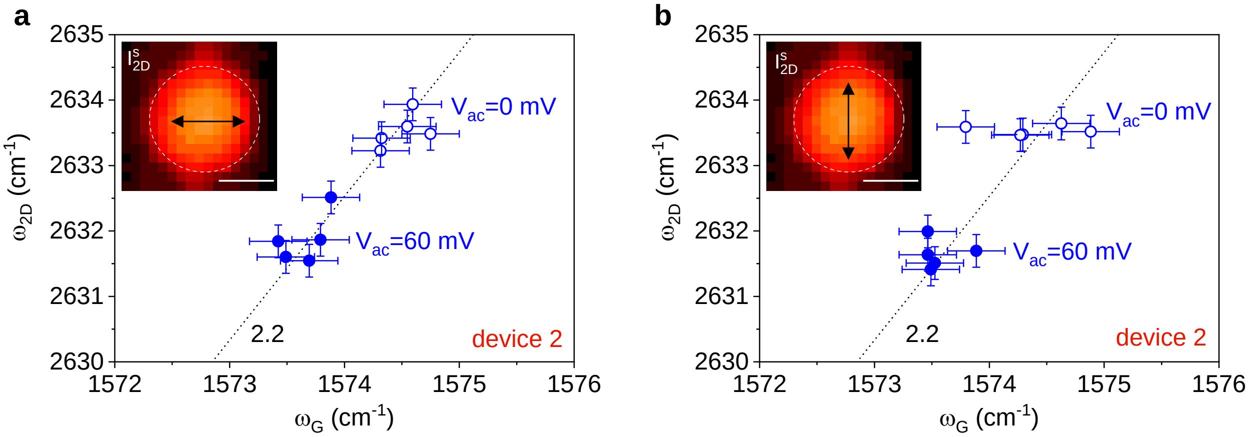

Supplementary Figure 20. Correlation plot of the frequencies of the G-mode and 2D-mode frequencies in device 2. The plots in **a** and **b** are made from the data in Supplementary Fig. **19** and in Fig. 4, respectively. The dashed lines with a slope of 2.2 are guides to the eye showing the expected correlation in the case of strain-induced phonon softening. See also Figure 4 in the main text and related discussion.

Supplementary Note 11. Supplementary data on device 3

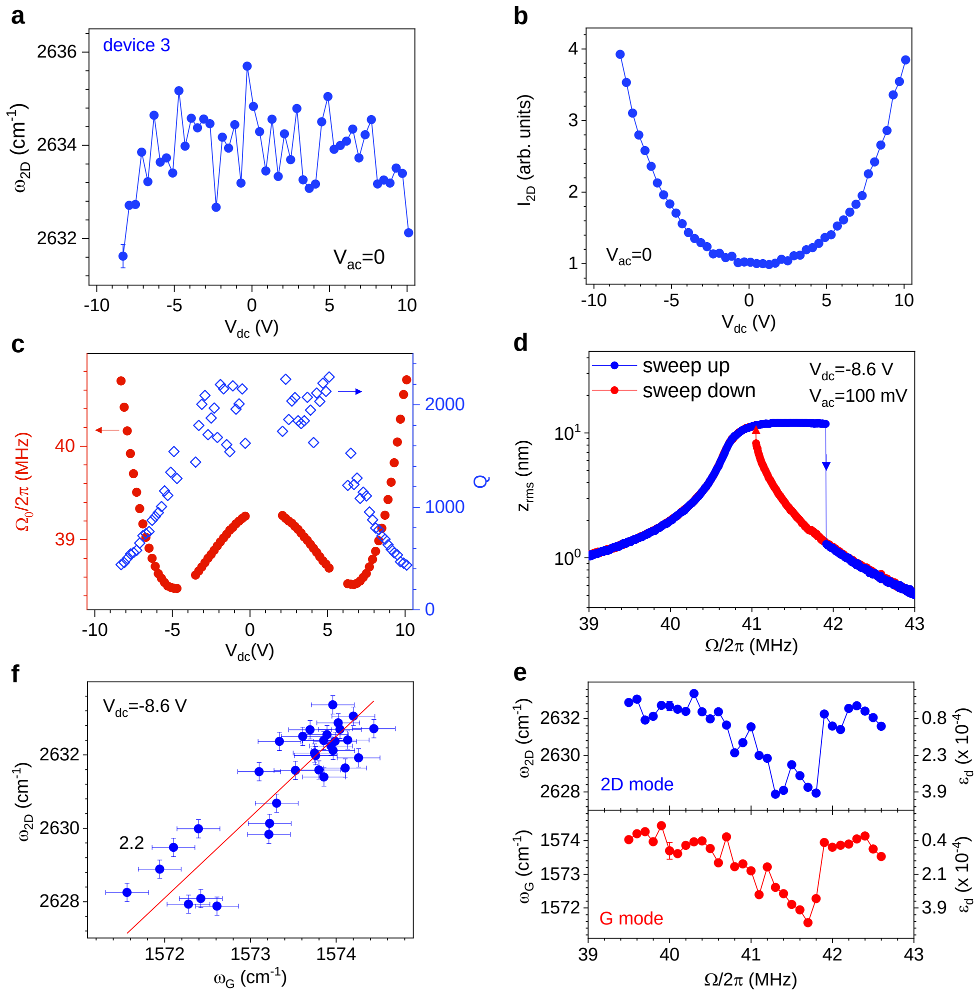

Supplementary Figure 21. **Dynamically-induced strain in device 3. a,b**, Frequency and Raman intensity of the 2D mode as a function of $V_{\rm dc}$ with $V_{\rm ac} = 0$ for another graphene drum (device 2). This device exhibits larger built in-tension ($\varepsilon_0 \approx 0.014\,\%$, estimated from Eq. (S12)) and thus reduced gate-tunability as compared to device 1. The limited tunability ($\sim 5\,\%$ over the range of $V_{\rm dc}$ explored here) is due to a negative spring effect that competes with the gate-induced tension, leading to a "W-shaped" characteristics[20,36,37]. **c**, Mechanical frequency and corresponding Q-factor as a function of $V_{\rm dc}$. **d**, Frequency-response curves at $V_{\rm dc} = -8.6$ V with $V_{\rm ac} = 100$ mV. **e,** Corresponding dynamically-induced G- and 2D-mode downshifts and estimated dynamical strain $\varepsilon_{\rm d}$. **f,** Correlation between the G- and 2D-mode frequencies. The straight black line with a slope of 2.2 is a guide to the eye showing the expected correlation for strain-induced phonon softening.

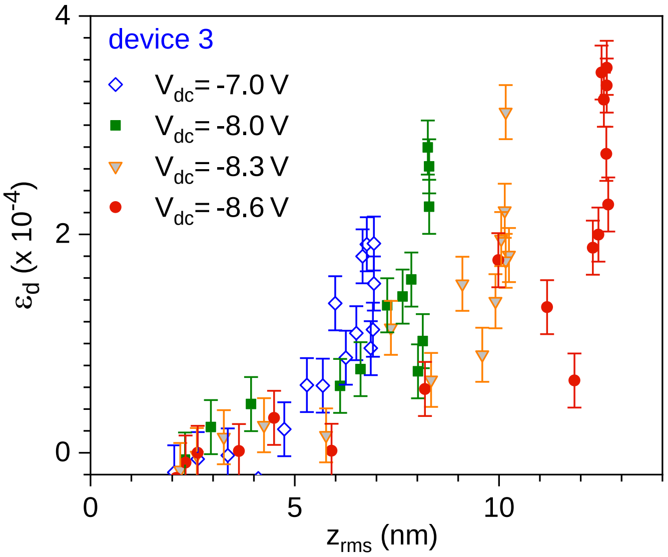

Supplementary Figure 22. **Gate-bias dependent dynamically-induced strain** $\varepsilon_{\rm d}$ **measured in device 3.** $\varepsilon_{\rm d}$ is obtained from the 2D-mode softening recorded during frequency sweeps (akin to Supplementary Fig. **21** and Fig. 3) and plotted as a function of $z_{\rm rms}$, for $V_{\rm dc} = -7$ V, -8.0 V, -8.3 V and -8.6 V. The error bars come from the standard deviation of the fits of the Raman spectra.

SUPPLEMENTARY REFERENCES

[1] Ferrari, A. C. & Basko, D. M. Raman spectroscopy as a versatile tool for studying the properties of graphene. Nat. Nanotechnol. **8**, 235–246 (2013).

[2] Froehlicher, G. & Berciaud, S. Raman spectroscopy of electrochemically gated graphene transistors: Geometrical capacitance, electron-phonon, electron-electron, and electron-defect scattering. Phys. Rev. B **91**, 205413 (2015).

[3] Maultzsch, J., Reich, S. & Thomsen, C. Double-resonant Raman scattering in graphite: Interference effects, selection rules, and phonon dispersion. Phys. Rev. B **70**, 155403 (2004).

[4] Basko, D. M. Theory of resonant multiphonon Raman scattering in graphene. Phys. Rev. B **78**, 125418 (2008).

[5] Venezuela, P., Lazzeri, M. & Mauri, F. Theory of double-resonant Raman spectra in graphene: Intensity and line shape of defect-induced and two-phonon bands. Phys. Rev. B **84**, 1–25 (2011).

[6] Berciaud, S. et al. Intrinsic line shape of the Raman 2D-mode in freestanding graphene monolayers. Nano Lett. **13**, 3517–3523 (2013).

[7] Chen, C.-F. et al. Controlling inelastic light scattering quantum pathways in graphene. Nature **471**, 617–620 (2011).

[8] Blake, P. et al. Making graphene visible. Applied Physics Letters **91**, 2007–2009 (2007).

[9] Davidovikj, D. et al. Nonlinear dynamic characterization of two-dimensional materials. Nat. Commun. **8**, 1253 (2017).

[10] Yoon, D. et al. Interference effect on Raman spectrum of graphene on SiO_2/Si. Phys. Rev. B **80**, 125422 (2009).

[11] Metten, D., Federspiel, F., Romeo, M. & Berciaud, S. All-optical blister test of suspended graphene using micro-Raman spectroscopy. Phys. Rev. Applied **2**, 054008 (2014).

[12] Metten, D., Froehlicher, G. & Berciaud, S. Monitoring electrostatically-induced deflection, strain and doping in suspended graphene using Raman spectroscopy. 2D Mater. **4**, 014004 (2016).

[13] Cattiaux, D., Kumar, S., Zhou, X., Fefferman, A. & Collin, E. Geometrical nonlinearity of circular plates and membranes. arXiv preprint arXiv:1910.02852 (2019).

[14] Koenig, S. P., Boddeti, N. G., Dunn, M. L. & Bunch, J. S. Ultrastrong adhesion of graphene membranes. Nat. Nanotechnol. **6**, 543–546 (2011).

[15] Androulidakis, C. et al. Graphene flakes under controlled biaxial deformation. Scientific Reports **5**, 18219.

[16] Metten, D., Froehlicher, G. & Berciaud, S. Doping- and interference-free measurement of i_{2d}/i_g in suspended monolayer graphene blisters. Phys. Status Solidi B **252**, 2390–2394 (2015).

[17] Nayfeh, A. H. & Mook, D. T. Nonlinear Oscillations (Wiley, 2007).

[18] Eichler, A., Moser, J., Dykman, M. I. & Bachtold, A. Symmetry breaking in a mechanical resonator made from a carbon nanotube. Nat. Commun. **4**, 2843 (2013).

[19] Hauer, B. D., Doolin, C., Beach, K. S. & Davis, J. P. A general procedure for thermomechanical calibration of nano/micro-mechanical resonators. Annals of Physics **339**, 181–207 (2013).

[20] Weber, P., Güttinger, J., Tsioutsios, I., Chang, D. E. & Bachtold, A. Coupling graphene mechanical resonators to superconducting microwave cavities. Nano Lett. **14**, 2854–2860 (2014).

[21] Nicholl, R. J. et al. The effect of intrinsic crumpling on the mechanics of free-standing graphene. Nat. Commun. **6**, 8789 (2015).

[22] Nicholl, R. J. T., Lavrik, N. V., Vlassiouk, I., Srijanto, B. R. & Bolotin, K. I. Hidden Area and Mechanical Nonlinearities in Freestanding Graphene. Phys. Rev. Lett. **118**, 266101 (2017).

[23] Bolotin, K. I. et al. Ultrahigh electron mobility in suspended graphene. Solid State Commun. **146**, 351–355 (2008).

[24] Berciaud, S., Ryu, S., Brus, L. E. & Heinz, T. F. Probing the lntrinsic properties of exfoliated graphene: Raman spectroscopy of free-standing monolayers. Nano Lett. **9**, 346–352 (2009).

[25] Berciaud, S., Potemski, M. & Faugeras, C. Probing electronic excitations in mono- to pentalayer graphene by micro magneto-raman spectroscopy. Nano Lett. **14**, 4548–4553 (2014).

[26] Davidovikj, D. et al. Visualizing the Motion of Graphene Nanodrums. Nano Lett. **16**, 2768–2773 (2016).

[27] In principle, Eq. (S9) could include other non-linear contributions, and in particular a non-linear damping term ($\propto \dot{z}z^2$)[38,39]. Non-linear damping may broaden the frequency-dependent mechanical susceptibility of our drums, reduce its resonant amplitude and may thus act against the enhancement of $\varepsilon_{\rm d}$. As a result, more pronounced dynamically-induced strain enhancement could be achieved provided non-linear damping in minimized.

[28] Schwarz, C. Optomechanical, Vibrational and Thermal Properties of Suspended Graphene Membranes. PhD dissertation, Institut Neel, Grenoble (2016).

[29] Lee, C., Wei, X., Kysar, J. W. & Hone, J. Measurement of the Elastic Properties and Intrinsic

Strength of Monolayer Graphene. Science **321**, 385–388 (2008).

[30] Schmid, S., Villanueva, L. G. & Roukes, M. L. Fundamentals of nanomechanical resonators (Springer, 2016).

[31] Yang, F. et al. Spatial modulation of nonlinear flexural vibrations of membrane resonators. Phys. Rev. Lett. **122**, 154301 (2019).

[32] Sajadi, B. et al. Experimental characterization of graphene by electrostatic resonance frequency tuning. Journal of Applied Physics **122**, 234302 (2017).

[33] Calizo, I., Balandin, A. A., Bao, W., Miao, F. & Lau, C. N. Temperature dependence of the Raman spectra of graphene and graphene multilayers. Nano Lett. **7**, 2645–2649 (2007).

[34] Barton, R. A. et al. Photothermal Self-Oscillation and Laser Cooling of Graphene Optomechanical Systems. Nano Lett. **12**, 4681–4686 (2012).

[35] Yoon, D., Son, Y.-W. & Cheong, H. Negative thermal expansion coefficient of graphene measured by raman spectroscopy. Nano Lett. **11**, 3227–3231 (2011).

[36] Singh, V. et al. Probing thermal expansion of graphene and modal dispersion at low-temperature using graphene nanoelectromechanical systems resonators. Nanotechnology **21**, 165204 (2010).

[37] Lee, J. et al. Electrically tunable single- and few-layer MoS 2 nanoelectromechanical systems with broad dynamic range. Science Advances **4**, eaao6653 (2018).

[38] Eichler, A. et al. Nonlinear damping in mechanical resonators made from carbon nanotubes and graphene. Nat. Nanotechnol. **6**, 339–342 (2011).

[39] Imboden, M., Williams, O. & Mohanty, P. Nonlinear dissipation in diamond nanoelectromechanical resonators. Appl. Phys. Lett. **102**, 103502 (2013).
